# Stochastic Individual-Based Modeling of Bacterial Growth and Division Using Flow Cytometry

**DOI:** 10.3389/fmicb.2017.02626

**Published:** 2018-01-05

**Authors:** Míriam R. García, José A. Vázquez, Isabel G. Teixeira, Antonio A. Alonso

**Affiliations:** ^1^Bioprocess Engineering Group, Marine Research Institute-Spanish National Research Council (IIM-CSIC), Vigo, Spain; ^2^Group of Recycling and Valorisation of Waste Materials, Marine Research Institute-Spanish National Research Council (IIM-CSIC), Vigo, Spain; ^3^Oceanology, Marine Research Institute-Spanish National Research Council (IIM-CSIC), Vigo, Spain

**Keywords:** individual-based modeling, stochastic modeling, cell cycle, bacterial growth and division, modified Fokker-Planck equation, flow cytometry, coccoid bacteria, predictive microbiology

## Abstract

A realistic description of the variability in bacterial growth and division is critical to produce reliable predictions of safety risks along the food chain. Individual-based modeling of bacteria provides the theoretical framework to deal with this variability, but it requires information about the individual behavior of bacteria inside populations. In this work, we overcome this problem by estimating the individual behavior of bacteria from population statistics obtained with flow cytometry. For this objective, a stochastic individual-based modeling framework is defined based on standard assumptions during division and exponential growth. The unknown single-cell parameters required for running the individual-based modeling simulations, such as cell size growth rate, are estimated from the flow cytometry data. Instead of using directly the individual-based model, we make use of a modified Fokker-Plank equation. This only equation simulates the population statistics in function of the unknown single-cell parameters. We test the validity of the approach by modeling the growth and division of *Pediococcus acidilactici* within the exponential phase. Estimations reveal the statistics of cell growth and division using only data from flow cytometry at a given time. From the relationship between the mother and daughter volumes, we also predict that *P. acidilactici* divide into two successive parallel planes.

## 1. Introduction

Population- and individual-based modeling are usually presented as incompatible approaches, although both describe the same system at different levels (Fahse et al., 1998; Wilson, 1998).

Traditionally, deterministic population-based models have been the underlying method behind predictive microbiology (Baranyi and Roberts, 1995). These models have been successfully applied to, for example, monitoring of food spoilage and microbial safety (Koutsoumanis and Nychas, 2000; Ross et al., 2000), smart sensing of food quality (García et al., 2015, 2017), Quantitative Microbial Risk Assessment (Cassin et al., 1998; Membré and Lambert, 2008), and design and control of food processes (Simpson et al., 1993; Alonso et al., 2013).

Over the last 15 years, stochastic individual-based modeling emerged as a promising tool to produce realistic estimations of safety risks along the food chain by describing the variability of single-cell behavior and small populations (Ferrer et al., 2009; Augustin et al., 2015; Koutsoumanis and Aspridou, 2017). Often, contamination of food starts with a small number of bacteria that adapt and proliferate on a given food matrix. At low cell concentrations, standard deterministic population models fail to predict the variability of the bacterial population. This is so because, at low initial cell numbers, heterogeneity between individuals and its influence on the division times become relevant and have a net influence on the population. Consequently, the behavior of individual cells cannot be neglected when assessing possible health risks along the food chain, either during storage or during distribution.

There are still many challenges in individual-based modeling, including the lack of information about single-cell behavior inside a population. The emergence of individual-based modeling was possible thanks to two main factors: (1) the increase of computer processing power and (2) the availability of single-cell measurements using new techniques such as the “mother machine” microfluidic device (Wang et al., 2010). However, the information from single-cell measurements is limited in those techniques where cells have to be isolated from the population. That was illustrated for example by Gangan and Athale (2017), who showed the difference in single-cell growth in a “mother machine” or in a population.

In this work, we hypothesize that population statistics of cell volume encode information about single-cell growth and division that can be used for individual-based modeling. For this purpose, we derive a modified Fokker-Planck Equation (forward Kolmogorov equation) describing the population volume distribution. The underlying idea is similar to that in Alonso et al. (2014) who derived a backward Kolmogorov equation to estimate single-cell growth using time-to-division distributions. We should remark that not only our approach is different, but it extends the previous theory to consider not only single-cell growth but also single-cell division. This allows us to simulate the individual-based modeling of bacterial growth and division.

In the first part of the work, we will test theoretically how population statistics obey a modified Fokker-Planck equation that encodes single-cell information. The equivalent individual-based modeling approach is derived in parallel to check consistency. Both models simulate single-cell growth and division assuming that cell volumes grow exponentially and cells divide following the sizer principle, i.e., division occurs at a critical volume (Métris et al., 2005; Alonso et al., 2014; Robert et al., 2014). Whereas, exponential growth of cell volume is a standard principle in bacterial physiology (Fishov et al., 1995), the main trigger of bacterial division is still a matter of controversy (Taheri-Araghi et al., 2015). There are three major paradigms: the sizer, timer, and adder principle depending on whether division is triggered by a certain volume, time, or after growing a given volume. As in most predictive microbiology studies (Métris et al., 2005; Alonso et al., 2014), we focus on fully adapted cells (medium growth is kept constant and measurements are within the exponential phase) and the sizer principle remains the reasonable assumption.

Once the theory is established, we combine the modified Fokker-Planck equation with flow cytometry data to find the single-cell behavior of *Pediococcus acidilactici* within the exponential phase. The food industry is interested in this species for several reasons, including its probiotic attributes (Planas et al., 2004; Standen et al., 2015), its ability to valorize food wastes (Vázquez et al., 2011; Banwo et al., 2013; Scatassa et al., 2015), and its ability to produce a very potent and broad-spectrum bacteriocin (pediocin SA-1) with high capacities as food biopreservative (Ray, 1992; Anastasiadou et al., 2008; Vázquez and Murado, 2008). *P. acidilactici* has been also selected for being coccoid cells with interest in the food industry. This shape makes easier to find correlations between side scatter and volume, and differ from the well-studied *Escherichia coli* (model of rod-shaped cells). We should stress that cell volume, membrane area and diameter scale similarly when the cell is rod-shaped, although that is not the case for round cells. For such reason, along this work we consider the term size as equivalent to volume, but not bacterial diameter or membrane area.

## 2. Materials and methods

This work combines theory with experimental data and requires three types of methodologies to (1) develop models at the single-cell and population level, (2) acquire data with flow cytometry and optical density, and (3) determine the best parameters to reconcile the theory with the experimental results.

### 2.1. Modeling at single-cell and population levels

#### 2.1.1. Individual-based modeling of single-cell bacterial growth and division

We tested different alternatives with stochastic or deterministic division and growth, that are specific cases of the general individual-based modeling approach we describe in this section.

The model assumes that the growth of the logarithm of the single-cell volume is subject to a stochastic fluctuation δ*W* characterized by a Wiener process (Alonso et al., 2014):

(1a) $$\begin{array}{c} \delta X^{i}=\mu \delta t+\xi \delta W\text{ with }X^{i}=\text{ln }(V^{i}) \end{array}$$

where *X*^*i*^ represents the volume of cell *i* (*V*^*i*^) in a logarithmic scale, μ represents the growth rate within the exponential phase and ξ is the intensity of the stochastic fluctuation. For the case of deterministic division ξ = 0 and cell volumes grow exponentially.

The division was modeled by adding a new cell to the population and resizing mother and newborn cell to the daughter size. The division event is triggered when the size of one or more cells is greater than a continuous random variable *X*_*m*_ with statistics defined by the probability density function (pdf) of mother sizes (*f*_*X*_*m*__(*x*)):

(1b) $$\begin{array}{ccc} \text{If }X^{i}\geq X_{m}\sim f_{X_{m}}(x), & X^{n+1} & \to X^{i}-\text{ln}(2) \end{array}$$

(1c) $$\begin{array}{cc} X^{i} & \to X^{i}-\text{ln}(2) \end{array}$$

where *n* is the number of cells in the population, *i* runs from 1 to *n* and the daughter volume is half the mother volume (υ_*d*_ = υ_*m*_/2).

We tested different probability density functions to describe the statistics of cell division, i.e., for the probability density function of mother sizes *f*_*X*_*m*__(*x*). The probability showing the best agreement with the data suggests that the volume of the mothers *V*_*m*_ is a random variable following a log-normal distribution (Koch, 1966; Amir, 2014):

(1d) $$\begin{array}{c} f_{X_{m}}(x)=N(x_{m},\sigma^{2})\text{ with }x_{m}=\text{ln}(\upsilon_{m}) \end{array}$$

For simulating the deterministic division, σ was set to zero so that the normal distribution turns into Dirac delta function centered at the logarithm of the mother size *x*_*m*_.

Simulations were initialized with a single cell ($X^{1}=x_{d}=\ln\left(\upsilon_{d}\right)$) and run for a given time horizon where a given cell and its offspring grow following (1a) and divide according to the rule in (1b). We selected the Euler-Maruyama algorithm to solve the stochastic differential equations for its simplicity as compared with other numerical methods (Higham, 2001). The bins of all the histograms to represent the population statistics were determined using the Freedman-Diaconis rule (Freedman and Diaconis, 1981). For convenience, simulations of population dynamics from the proposed single-cell stochastic model were performed on a cluster composed of 12 processing nodes (openSUSE 11.0 Linux with 23.5 GB of RAM) and 160 processors in total, using the SGE task manager to distribute the calculations among them.

#### 2.1.2. Population modeling using the modified fokker-planck equation

The statistic of the cell sizes in the population is formally described by a probability density function (pdf) *p*(*t, x*) that depends on size *x* and time *t*. For the sake of clarity, we keep previous subsection notation: υ and *x* denote size in terms of volume and natural logarithm of the volume, respectively. Subindexes *d* and *m* denote daughter and mother respectively, whereas *f*_*X*_*m*__ and *f*_*X*_*d*__ are the corresponding pdfs for sizes. As before, growth rate and fluctuation intensity are denoted by μ and ξ, respectively. The function *p*(*t, x*) is the solution of the following modified Fokker-Planck equation:

(2a) $$\frac{\partial p(t,x)}{\partial t}=\underset{\text{cell growth}=\frac{\partial J(t,x)}{\partial x}}{\underset{︸}{\frac{\xi^{2}}{2}\frac{\partial^{2}p(t,x)}{\partial x^{2}}-\mu \frac{\partial p(t,x)}{\partial x}}}+\underset{\text{division}}{\underset{︸}{2f_{X_{d}}(x)Z-f_{X_{m}}(x)Z}}-\underset{\text{normalization}}{\underset{︸}{p(t,x)Z}}$$

being

(2b) $$\begin{array}{c} Z=\int_{\underline{x}}^{\bar{x}}F_{m}(x)\frac{\partial J(t,x)}{\partial x}dx \end{array}$$

(2c) $$\begin{array}{c} p(t,\underline{x})=p(t,\bar{x})=0\;\forall t\text{ boundary conditions} \end{array}$$

(2d) $$\begin{array}{c} p(0,x)=\delta (x-x_{d})\;\forall x\text{ initial conditions} \end{array}$$

where $f_{X_{d}}\left(x\right)=N\left(x_{d},\sigma^{2}\right)$ is the pdf of daughter sizes, $f_{X_{m}}\left(x\right)=N\left(x_{m},\sigma^{2}\right)$ the pdf of mother sizes and *F*_*X*_*m*__ its cumulative distribution function. The model is valid only for large domains $x\in [\underline{x},\bar{x}]$ where no cell sizes are close to their (minimum and maximum) boundaries.

Without the terms of division and normalization, Equation (2a) is the classical Fokker-Planck equation of the stochastic differential Equation (1a). It explains how the change in the distribution of volumes depends on a diffusion term which is proportional to the square of the fluctuation plus a convective term proportional to the cell growth rate (Gardiner, 2004; Alonso et al., 2014). To account for division, we have added two terms proportional to the pdfs of daughter and mother volumes. Normalization is required for *p*(*t, x*) to be a pdf. This is performed via the last term in the right hand side.

Simulations were performed using the finite difference discretization scheme in http://www.matmol.org/ (Vande Wouwer et al., 2014) for the *x* domain. For all cases, the discretization scheme consisted of 501 elements. That was considered enough to approximate the equation since further refinements resulted in negligible improvements in the accuracy of the results. First derivatives were calculated using an upwind 5 points in the stencil and second derivatives with centred 5 points in the stencil. Due to the hard non-linearity at division, not only is a refined mesh in *x* required, but also a stiff time integrator. Ode15s in Matlab (Shampine and Reichelt, 1997) was selected for time integration of the resulted set of ordinary differential equations after the spatial discretization.

### 2.2. Data acquisition and analysis

#### 2.2.1. Microbiological methods

*Pediococcus acidilactici* NRRL B-5627 was kindly provided by the Northern Regional Research Laboratory (Peoria, IL, USA). Stock cultures of bacterium were stored at -80 °C in MRS commercial medium (Pronadisa, Hispanlab S.A., Spain) with 25% glycerol. The inoculum to study the growth dynamics of *P. acidilactici* was prepared as follows:

1. One hundred and fifty milliliters of cellular suspension from the cryotube was transferred to 5 mL of MRS fresh medium and then incubated at 30 °C in an orbital shaker at 200 rpm for 16 h.
2. From the obtained culture, 1 mL was added to an Erlenmeyer flask with 150 mL of MRS fresh medium and fermented at 30 °C/200 rpm for 22 h.
3. From the previous cultivation, serial 10-fold dilutions were prepared in peptone-buffered solutions, and 0.1 mL samples were plated (MRS agar medium) in triplicate and incubated at 30 °C for 48 h.

Five individual colonies from plates were isolated and transferred to 5 Erlenmeyer flasks with 200 mL of MRS fresh medium and cultivated at 30 °C/200 rpm. Samples from flasks were taken each hour up to 17 h (except at 12 and 16 h). All samples were separated in two aliquots, one of them was prepared for cytometer evaluation following the indications described in the next section. The other aliquot was centrifuged at 4,000 g for 15 min and the sediment washed twice and re-suspended in distilled water at an appropriate dilution to measure the optical density at 700 nm. The dry weight was estimated from a calibration curve ($G\left(g/L\right)=-0.008+0.342A_{700}+0.028A_{700}^{2}$).

The percentage of viable cells smaller than certain diameters was calculated during the exponential phase. The cultures at 8 h were filtered, under sterile conditions, through 1.2 and 1 μm glass microfiber filters (Filter-Lab, Filtros Anoia S.A., Barcelona, Spain). Thus, in these filtered solutions and in the final unfiltered culture (control), viable cells (colony forming units per mL) were quantified by count on MRS-agar plate as it was mentioned in the previous paragraph.

#### 2.2.2. Flow cytometer data acquisition

The abundance and size of *P. acidilactici* were determined with a BD FACSCalibur flow cytometer (BD Biosciences, San José, CA, USA) equipped with a laser emitting at 488 nm. Bacteria samples were fixed with a P+G solution (1 % paraformaldehyde + 0.05 % glutaraldehyde) at 10 % final concentration for 15 min in the dark. Then, samples were quickly frozen in liquid nitrogen and stored at -80 °C. Prior to analysis, bacteria were stained with SybrGreen I DNA dye (5 mM final concentration) and diluted adequately. Bacteria were detected in the flow cytometer by their signature in a plot of Side Scatter (SSC) vs. FL1 (green fluorescence). All the reagents and chemicals were purchased from Sigma-Aldrich S.A. (St. Louis, MO, USA).

#### 2.2.3. Estimation of *P. acidilactici* population growth

The logistic equation was used to fit the population growth data (Zwietering et al., 1990; Peleg and Shetty, 1997). Biomass and number of cells were obtained respectively by dry weight and cytometry of *P. acidilactici*:

(3) $$\begin{array}{c} G=\frac{G_{m}}{1+\exp\left[2+\mu^{p}(\lambda -t)\right]} \end{array}$$

where *G* is the *P. acidilactici* growth as biomass or cells (g L^−1^ or cells mL^−1^). *G*_*m*_ represents the maximum growth or plateau phase (g L^−1^ or cells mL^−1^), λ is the lag phase (h), *t* denotes the time of culture (h) and $\mu^{p}=\frac{4\mu_{m}^{p}}{G_{m}}$ is the specific growth rate of the population (h^−1^) with $\mu_{m}^{p}$ being the maximum specific growth rate (g L^−1^ h^−1^ or cells mL^−1^ h^−1^).

Non-linear least-squares method (quasi-Newton) was applied for growth data modeling. Confidence intervals from the parametric estimates (Student's *t*-test) and consistency of mathematical models (Fisher's *F*-test) and residual analysis (Durbin-Watson test) were evaluated by “SolverAid” macro (Levie's Excellaneous website: http://www.bowdoin.edu/~rdelevie/excellaneous)

### 2.3. Model calibration

Reliable single-cell parameters of *P. acidilactici* are unknown and were estimated by minimizing the distance between the modified Fokker-Planck Equations (2a–2d) and the data from the flow cytometry. The unknown parameters are the growth rate μ, the fluctuation intensity ξ, the statistics of the mother distribution (*x*_*d*_ and σ) and the parameter relating mother and daughter sizes ω. The estimated parameters were used to simulate single-cell dynamics based on the individual-based modeling approach (1a–1d).

The method of least squares was used to define the distance between the model and the data. Essentially, it aims at minimizing the differences between the stationary distribution of sizes calculated with (2a–2d) and the stationary distribution estimated from the data. The experimental distribution was obtained using different replicates at one given time using histograms with a number of bins given by the Freedman-Diaconis rule. For noisy data, optimization could lead to a multimodal problem, i.e., it has several sub-optimal solutions (Vilas et al., 2017). To assure convergence to the global solution in a reasonable time, the global optimizer Enhanced Scatter Search (eSS) was employed (Egea et al., 2009).

## 3. Results and discussion

### 3.1. Population statistics of single-cell growth and division obey a modified fokker-planck equation

#### 3.1.1. Deterministic growth and division

We first simulate the individual-based modeling of deterministic growth and division. We assume that cell volumes grow exponentially and cells divide following the sizer principle, i.e., division occurs at a critical volume (Métris et al., 2005; Alonso et al., 2014; Robert et al., 2014). The adder model is more realistic while cells are adapting to the growth media (lag phase), but it becomes a sizer when, as in our case, cells are fully adapted within the exponential phase (see Figure 1B in Sauls et al., 2016). It should be noted that considering an adder model would complicate considerably the derivation of the equivalent Fokker-Planck equation without altering the final results.

**Figure 1:**
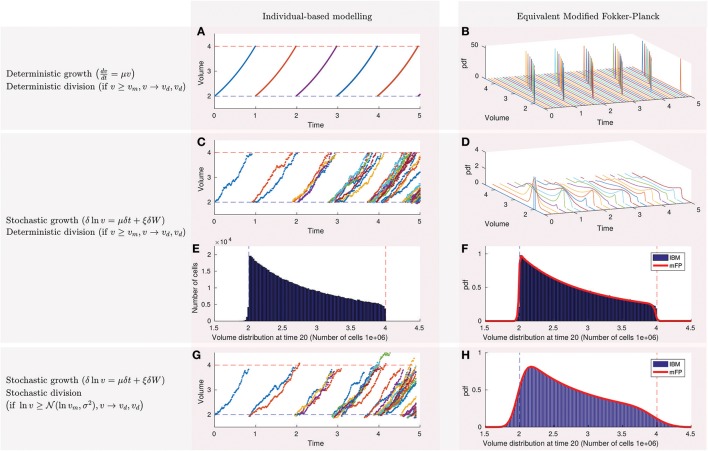
Population statistics of bacterial growth obey a modified Fokker-Planck equation from which we can extract information about single-cell behavior. **(A)** Simulation of the deterministic individual-based modeling approach shows that cells grow exponentially at the same rate and divide at the same time into two cells with equal volume when reaching a critical size (the mother volume). **(B)** The statistics of the population volume obey a modified Fokker-Planck equation that oscillates between mother and daughter volumes without reaching a stationary distribution. **(C)** Individual-based modeling with stochastic growth assumes that the logarithm of the volume is subject to a stochastic fluctuation δ*W* characterized by a Wiener process. **(D)** Simulation of the equivalent modified Fokker-Plank now shows that the population volumes evolve to a stationary distribution. **(E)** Individual-based modeling can be used to estimate the histogram of the stationary distribution, but at the expenses of expensive computations that scale exponentially with time and linearly with the number of cells in the population. The distribution is sharp and skewed to the right and encodes single-cell features such as the mother and daughter volumes, fluctuation, and growth ratios. **(F)** The modified Fokker-Planck simulates the continuous shape of the stationary distribution (red line) in a efficient way that is independent on the number of cells within the population. **(G)** The individual-based modeling simulates stochastic growth and division. **(H)** The resulting stationary distribution of the population volumes is smooth and equivalent when calculated using the individual-based modeling and the modified Fokker-Planck equation.

Figure 1A shows the simulations of the deterministic single-cell dynamics. All the cells have the same volume because they grow and divide at the same velocity and time. For this example only 3 parameters are required: the daughter volume (υ_*d*_) and the mother volume (υ_*m*_ = 4), depicted in dashed blue and red lines, and the single-cell growth rate μ = 0.7. At time 0 there is only one cell that grows until reaching the critical volume of division (or mother volume). This cell divides into two cells of half their volumes (daughter volume). Therefore, at time 1 there are two cells that cannot be distinguished in the figure because their dynamics overlap. The process is repeated until reaching a population of 32 cells at time 5.

The dynamics of the population volume distribution obey a modified Fokker-Planck equation that, as shown in Figure 1B, consists of a pulse that oscillates between the daughter and mother volumes. Each color line represents the distribution at a different time and are simulated using the partial derivative Equation (2a) with stochastic parameters set to zero (ξ = 0 and σ = 0) and the single-cell parameters in Figure 1A (υ_*d*_ = 2, υ_*m*_ = 4, μ = 0.7).

This model with deterministic growth and division is invalid since the population statistics fluctuates instead of evolving to a stationary distribution. Fishov et al. (1995) explained how balanced exponential growth implies steady-state growth and stationary frequency distribution of the various components that constitute the cell. In words by Painter and Marr (1968) “the distribution of each intensive random variable is time-invariant.”

#### 3.1.2. Stochastic growth and deterministic division

Single-cell measurements of bacteria suggest that growth is a stochastic process (see for example Figure 1A in Deforet et al., 2015 for *Pseudomonas aeruginosa*). Alonso et al. (2014) proposed an individual-based modeling approach reproducing such single-cell dynamics. They assumed that the logarithm of the volume is subject to a stochastic fluctuation δ*W* characterized by a Wiener process following Equation (1a). We use the same assumption to model stochastic growth. Figure 1C shows simulations of the single-cell dynamics with stochastic growth and deterministic division. After the first division (time = 0.9) the dynamics of the two daughter volumes differ and the same happens with division times.

We confirm how simulations of the population statistics with stochastic growth now evolve to a stationary distribution (Figure 1D) as predicted by (Fishov et al., 1995). At time 0 the distribution is a Dirac delta function that spreads and moves between mother and daughter volumes. The rate of spread depends on the intensity of the fluctuation ξ whereas the velocity of the moving pulse is determined by the growth parameter μ. Single-cell parameters are as in previous section (υ_*d*_ = 2, υ_*m*_ = 4, μ = 0.7) except for the fluctuation that it is now ξ = 0.1.

The stationary distribution can be calculated either using a large number of single-cell simulations (1a–1d) or solving the modified Fokker-Planck equation (2a–2d). We plot in Figure 1E the histogram for a population of 1e6 cells using the individual-based modeling approach. The mode of the histogram coincides with the daughter volume whereas the end of the histogram is the mother volume. The shape depends on the growth rate μ and the fluctuation ξ. Normalizing the area of this histogram we obtain the blue probability density function in Figure 1F. The modified Fokker-Planck equation, on the other hand, calculates directly the probability density function (red line). Both approaches coincide as shown in Figure 1F.

Results with stochastic growth and deterministic division evolve to a stationary distribution, but sharper than observed experimentally. In fact, the distribution of *E. coli* is commonly approximated for some authors by a smooth log-normal distribution (Kaya and Koser, 2009; Athale and Chaudhari, 2011). In addition, the end of the distribution (the mother volume) is not exactly the double of the mode (the daughter volume) in experiments.

#### 3.1.3. Stochastic growth and division

A great amount of works in the literature assumes stochastic division and focus on the distribution of mother or daughter sizes (Amir, 2014; Taheri-Araghi, 2015; Taheri-Araghi et al., 2015; Sauls et al., 2016). Some works measure symmetric distributions close to a normal distribution (Sauls et al., 2016), whereas others assumed asymmetric distributions. That is the case of Koch (1966) and Amir (2014) who concluded that the daughter volume distribution is log-normal.

We extended our model considering that cells divide stochastically with a certain probability, either normal (Sauls et al., 2016) or log-normal (Amir, 2014). In other words, we moved from a strict sizer model to a sizer model with stochastic division. For the simulations in Figure 1G we assumed log-normal division. Now cells may divide before or after reaching the volume of 4 (red dashed line). As the model was implemented in the logarithm of the volume *X*, the log-normal stochastic division in the logarithm becomes a normal probability with mean υ_*m*_ = 4 and a standard deviation that we assumed to be σ = 0.1. The remaining parameters are kept as in the previous section (υ_*d*_ = 2, *v*_*m*_ = 4, μ = 0.7, ξ = 0.1).

As shown in Figure 1H, results from the individual-based modeling coincide with the modified Fokker-Planck also for stochastic growth and division.

We should note how it is critical to assume stochastic growth and division to obtain realistic and smooth stationary distributions where the mode is larger than the daughter volume (υ_*d*_).

#### 3.1.4. Comparison of individual-based modeling and population modeling with the modified fokker-planck equation

Individual-based modeling is a bottom-up approach providing valuable information at the single-cell level, but it requires parameters that cannot be easily measured (Ferrer et al., 2009; Augustin et al., 2015). The modified Fokker-Planck equation here presented focuses on population statistics that can be measured by flow cytometry. Comparisons between this equation and the experimental data are sufficient to estimate single-cell parameters that can be used for individual-based modeling.

The modified Fokker-Planck equation directly provides the evolution of the volume distribution without the need of predefining a probability density function. When populations are not large enough, the histograms calculated with individual-based modeling are too poor to extract relevant statistics. It is then usually preferred to assume a certain family of probability density functions. The precision of the modified Fokker-Planck equation, however, depends only on the discretization method to solve the partial differential equation, and works for large and small populations whenever the assumptions of the Wiener process are satisfied (Gardiner, 2004).

In addition, individual-based modeling is characterized by requiring long computational times which make its use prohibitive in applications that demand many model evaluations (An et al., 2017), such as parameter estimation. The computation time of individual-based modeling grows exponentially with time, whereas the growth is linear for the equivalent modified Fokker-Planck equation. Note that the individual-based modeling approach requires one equation per cell. As cells grow exponentially, computation time scales linearly with the number of cells and exponentially with time. The modified Fokker-Planck equation, however, is a unique partial differential equation (PDE). Its computation time will depend on the degree of discretization and the simulation time. For the examples in Figure 1, computational times (2–6 s) are similar until time 15 (population of less than 3e4 cells) for both approaches. However from this time the modified Fokker-Plank equation becomes more efficient in orders of magnitude.

Moreover, the partial differential equation for stochastic growth has a diffusion term allowing efficient simulations using the appropriate techniques. Classical discretization methods transform the partial differential equation into a large number of ordinary differential equations. When the original equation is diffusive, a number of methods are at hand to take advantage of this property and significantly reduce the number of ordinary differential equations, thus reducing computational times (Trefethen, 2000; García et al., 2008).

### 3.2. Flow cytometry allows estimation of volume distributions for *P. acidilactici*

**Figure 2:**
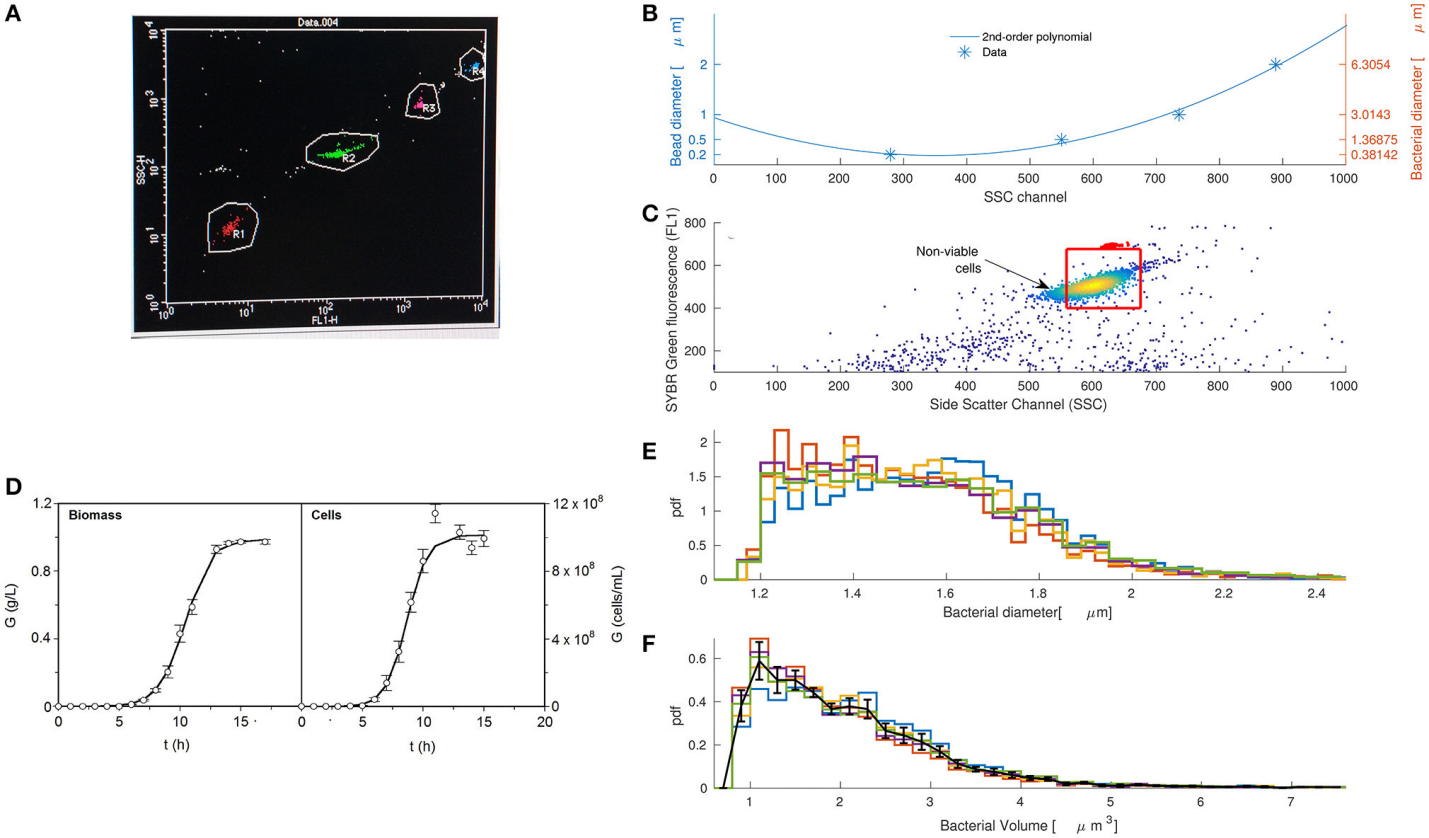
Flow cytometry is an efficient technique to extract volume distributions of coccoid bacteria such as *Pediococcus acidilactici*. **(A)** Side scatter light (y-axis) discriminates among round beads of different diameters represented in red, green, pink, and blue for diameters 0.2, 0.5, 1, and 2 μ*m*, respectively. **(B)** Bead diameter correlates with side scatter as a second order polynomial (Julià et al., 2000; Prats et al., 2010) and can be transformed into bacterial diameter following the linear relationship in Chandler et al. (2011). **(C)** Flow cytometry correlogram and gating (red box) of fluorescent dye Sybrgreen with side scatter light for *Pediococcus acidilactici* after 8 h of growth. **(D)** Growth kinetics of *P. acidilactici* in terms of biomass and cell counting shows that the selected time where we took the sample (*t* = 8 h) is within the exponential phase (error bars are the confidence of intervals for *n* = 5 and α = 0.05). **(E)** Estimated diameter distribution of five replicas of the population of *Pediococcus acidilactici* at time 8. **(F)** Population volume distributions of *Pediococcus acidilactici* at time 8.

Flow cytometry is the standard technique for fast acquisition of population statistics. It is commonly employed to estimate different mammalian cell characteristics such as cell size using forward scattered light. This technique is also useful for bacteria, but as their diameters are close to the light wavelength (488 nm or 0.5 μm), side scattered light has better resolution and is preferred. Figure 2A shows how side scattered light (y-axis) discriminates among different bead diameters. Events in red, green, pink and blue correspond with beads of diameters 0.2 , 0.5 , 1 , 2 μm, respectively. Sizes smaller than 0.2 μm were below the detection limit of the device.

Estimation of bacterial diameters from side scattered light requires two steps: (1) to find the correlation between the bead diameter and side scattered light and (2) to transform the bead diameters to bacterial diameters. Figure 2B shows that the bead diameter is a second order polynomial of side scatter (Julià et al., 2000; Prats et al., 2010). However, bacteria and polystyrene beads have different refractive indexes. To correct the differences in the refractive index we make use of the linear relationship in Chandler et al. (2011). The figure shows in the right y-axis the final relationship between bacterial diameter (d) and side scatter (SSC), outlined in the following expression:

(4) $$\begin{array}{c} d=a(p_{1}SSC^{2}+p_{2}SSC+p_{3})+b \end{array}$$

where *SSC* is the side scatter channel, *a* = 3.2911 and *b* = −0.2769 are the parameters provided in Chandler et al. (2011) to correct the differences in the refractive index and $p=\left[p_{1},p_{2},p_{3}\right]=\left[6.13\times 10^{-06},-0.0043,0.94\right]$ the estimated parameters of the second-order polynomial.

We acquired and processed side scatter data of *P. acidilactici* at one sampling time after 8 h of growth. Figure 2C shows the flow cytometry correlogram of Sybrgreen fluorescence and side scatter for one of the replicates. Red points indicate beads used to count the number of events.

The red box defines the gating where viable cells lie. We used two sources of information to define the gating: Sybrgreen fluorescence and experiments counting viable cells at different diameters. Sybrgreen was helpful to determine those events with too low DNA material to be consistent with a viable cell (upper and lower horizontal lines of red box). They probably represent either dead cells from the lag phase that have lost some of their DNA material, or free DNA detected as an event. Only with this gating, most diameters were between 1 and 2.5 μm as reported in the literature (Holt, 1994). However, the first calibrations of the model suggested that smaller cells were not able to divide. We passed cells through a 1.2 μm filter and found that only about 2% of the cells were viable. Consequently, we did not consider cells smaller than 1.2 μm (left vertical line of the red box).

In order to assume that the selected sampling time (*t* = 8 h) was within the exponential phase of growth, we estimated growth kinetics in terms of biomass and cell counting. Figure 2D shows the experimental curves with the standard sigmoid growth pattern for lactic acid bacteria. Both cases are described by the logistic equation (3) (*R*^2^ = 0.993–0.999). *p*-values from Fisher's *F*-test show consistency and robustness of the logistic to appropriately describe these profiles (Table 1). It is noted that no autocorrelation was observed in the fittings (data not shown). All parameters were statistically significant (t-Student test). The production of biomass was 25–30% lower as compared to previous cultures (Vázquez and Murado, 2008) which may be due to the minimum inocula employed in the present study. Typically, inocula used for the production of bacteriocins from lactic acid bacteria including *P. acidilactici* are much more populated, reaching values of 10^5^−10^7^ cfu/mL and longer productive periods (Vázquez et al., 2008).

**Table 1:** Summary of the parameter values obtained from the fittings of *P. acidilactici* growths (biomass and cells production) to the logistic Equation (3).

**Parameters**	**Biomass**	**Cells**
*G*_*m*_	0.990 ± 0.021gL^−1^	(10.14 ± 0.72) × 10^8^cells mL^−1^
μ^*p*^	0.904 ± 0.021h^−1^	1.163 ± 0.394h^−1^
λ	8.23 ± 0.18h	6.72 ± 0.61h
$\mu_{m}^{p}$	0.224 ± 0.018gL^−1^h^−1^	(2.95 ± 0.93) × 10^8^ cells mL^−1^h^−1^
*R*^2^	0.999	0.993
*p*-values	<0.0001	<0.0001

*Statistical parameters R^2^ and p-values are also shown*.

Five data replicates at *t* = 8 h and the relationship in (4) were combined to estimate the volume distribution of *P. acidilactici*. Figure 2E shows the bacterial diameter distribution of different replicates at time 8 h calculated from relationship (4). Volume distributions in Figure 2F are calculated by applying the volume equation of a sphere to the diameter histograms. Error bars show the mean and standard deviation of the histogram values for the five replicates. The distribution is smooth and skewed to the right, as expected from the modeling analysis in section 3.1. A similar behavior was observed in volume distributions of *E. coli* growing inside a population (Gangan and Athale, 2017).

### 3.3. The modified fokker-planck equation is combined with flow cytometry data to find the single-cell behavior of *P. acidilactici*

The volume distributions of the *P. acidilactici* at time 8 h provide enough information to find the single-cell parameters of the modified Fokker-Planck equation. The problem consists in finding the best set of parameters of the modified Fokker-Planck equation that represents the experimental volume distributions by solving a least square problem.

Preliminary computations demonstrated the inability of the model in (2a–2d) to reproduce the data, suggesting that the volume of each daughter is approximately one-fourth of the mother volume, i.e., υ_*d*_ = (1/4)υ_*m*_. This hypothesis contradicts the principle of binary fission and had to be rejected because, if a mother gives two cells of this size, total volume would be destroyed and not conserved during division. This would imply that the specific growth rate of the population μ^*p*^ differs from the growth rate of the single-cell volumes μ, contradicting common observations in rod-shaped bacteria (Taheri-Araghi et al., 2015; Harris and Theriot, 2016). We could devise that this hypothesis, however, may be plausible in coccoid cells since cell volume, membrane area, and diameter scale differently. In fact, assuming that cells are spheres, simple calculations indicate that membrane area, instead of volume, is conserved if υ_*d*_ = (1/4)υ_*m*_. However, it is well-known that coccoid bacteria create membrane (septal growth) before division (Pinho et al., 2013; Monteiro et al., 2015), suggesting again, that volume is conserved.

It resulted that the data pointed out to another mechanism to explain the daughter volumes: *P. acidilactici*, like *Pediococcus pentosaceus* and other coccoid cells (Zhou et al., 2010; Pinho et al., 2013; Monteiro et al., 2015), have two planes of division (Turner et al., 2010). That means that cells undergo two consecutive divisions and, at the time-scales of interest, one mother divides into four daughters with fourth of the mother volume. Hence we derive the individual-based and population-based modeling approaches considering two planes of division (see Appendix).

**Figure 3:**
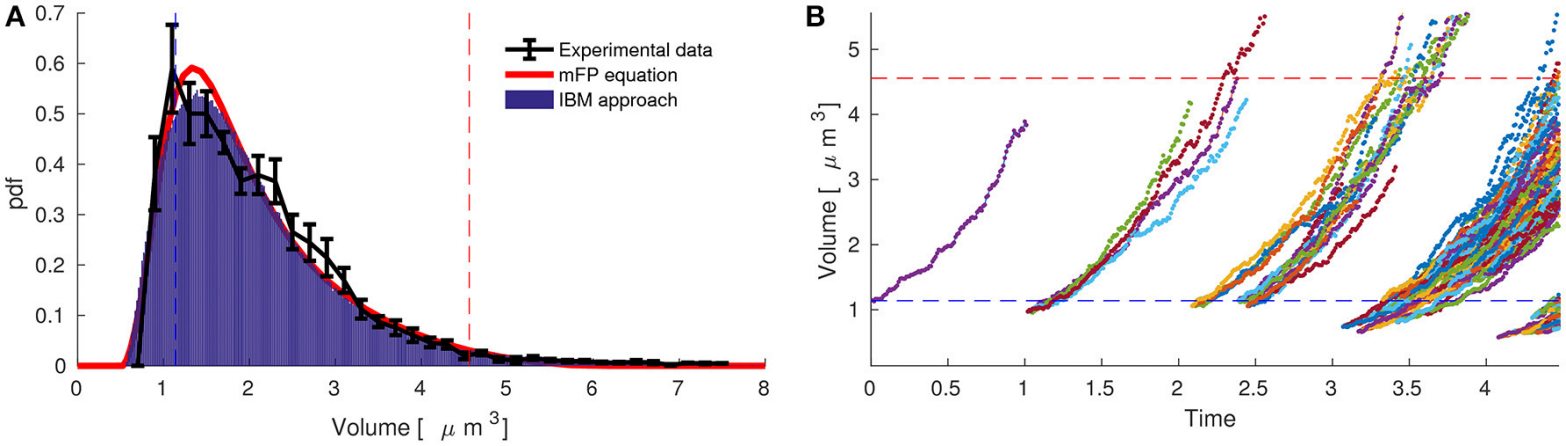
The modified Fokker-Planck equation allows us to estimate single-cell behavior of *Pediococcus acidilactici* from acquisition based on cytometry data at one sampling time within the exponential phase. **(A)** The stationary distribution of *Pediococcus acidilactici* (black line) coincides with the modified Fokker-Planck equation (red line) and the individual-based modeling (blue histogram). The single-cell parameters to simulate both models were obtained by minimizing the differences between data and the stationary Fokker-Plank equation. **(B)** Single-cell dynamics of *Pediococcus acidilactici* with the estimated parameters. During the cycle of a single-cell, the volume growths four times and it divides in four daughters.

Figure 3A compares experimental data with both models at the population and single-cell levels considering two planes of division. Table 2 shows the single-cell parameters used in the simulations. The three elements exhibit the same steady-state volume distribution. The black line represents the experimental data (see also Figure 2F) and the red line and histogram are the solutions of the modified Fokker-Planck distribution and individual-based modeling, respectively.

**Table 2:** Bounds and estimations of the best set of parameters of the modified Fokker-Planck equation to reproduce the experimental volume stationary distribution of *P. acidilactici*.

**θ**	**θ_min_**	**θ_max_**
υ_*m*_ = 4.5574	1	9
μ = 1.1619	0.7	1.5
ξ = 0.13439	0.075	0.2
σ = 0.3	0	0.3

*We assume that bacteria have 2 division planes and therefore one mother produces four daughters of 1/4 of the mother's volume. Upper bound on the standard deviation of the statistics of division σ was considered 0.3 to avoid overlapping between distribution of mother sizes and daughter sizes*.

All the estimated parameters are within the selected bounds (see Table 2) except for the standard deviation of the distribution of mother volumes (σ). Probably, this parameter tries to accommodate the errors for assuming that the mother divides into four perfectly round daughters. For coccoid cells with two planes of division, there is a short transition where cells are not completely round (Pinho et al., 2013; Monteiro et al., 2015). For the time-scales here considered this is a simplification that seems appropriate. Moreover, we have tried to reduce this bound resulting in similar estimations but with worse fit. For all these reasons we have considered an upper bound for the standard deviation of the mother distribution sufficiently small to avoid relevant overlapping between the mother and the daughter distributions. In this way, we force a scenario where the probability of having daughter volumes greater than mother volumes is low. Note that a standard deviation of 0.3 is reasonable attending to other mother distributions in the literature (Amir, 2014).

We also validate the results confirming that the specific growth rate of the population, μ^*p*^ in (3), coincides with the growth rate of the cell volume, μ in (1a) and (2a). Both population and volume should increase by one-fourth in one cell cycle. The specific growth rate of the population was estimated using the growth curves of *P. acidilactici* in Figure 2D. The calculated specific growth rate (1.1619 h^−1^) is similar to the rate using cell counting with cytometry (1.163 ± 0.394h^−1^) and larger than the rate estimated from the biomass growth curve (0.904 ± 0.021h^−1^).

Individual-based modeling of *P. acidilactici* can now be implemented using the estimated single-cell parameters in Table 2. Figure 3B depicts the simulations starting with one cell at the mean of the daughter volumes (υ_*d*_ = υ_*m*_/4 = 1.14). We observe how the fluctuations, dependent on ξ, resembles experimental single-cell dynamics in the literature (Deforet et al., 2015). In order to obtain such simulations, it is critical to consider that during division one mother gives four daughters, volume is conserved and that volumes grow four-fold during a cell-cycle.

## 4. Concluding remarks

In this work, we have developed a modified Fokker-Planck equation describing the statistics of a population from their single-cell parameters. The model is based on the assumptions that cell volumes grow exponentially and cells divide following the sizer principle. We have tested and numerically compared the modified Fokker-Planck with its equivalent individual-based modeling approach. Simulations resulted critical to understand several observed phenomena during the exponential phase of growth of bacteria, including the necessity of considering stochasticity to obtain a distribution of volumes that is time-invariant and similar to experimental observations.

The modified Fokker-Planck equation is also a powerful tool to estimate the behavior of single-cells inside populations. Instead of requiring single-cell measurements, we make use of flow cytometry to find the volume distribution of a population of *P. acidilactici* within the exponential phase. The combination of the modified Fokker-Planck equation with data provides information about the growth rate and stochasticity of the single-cell volume, as well as the statistics of the mother and daughter volumes. For a good correspondence between model and data, it is fundamental to assume that the *P. acidilactici* have two planes of division and its volume and population numbers grow by a fourth during a cell cycle.

The proposed methods allow efficient analysis of flow cytometry data to find single-cell behavior. In fact, they pave the way for studying cell heterogeneity in numerous applications in food microbiology, such as in quantitative risk assessment and prediction of shelf life.

## Author contributions

MG and AA: designed the theoretical study, developed the methodology, and drafted the manuscript; JV: designed the experimental study and analyzed the data; IT: collected the data in the flow cytometry; MG: conducted the modeling experiments. All authors approved the final version of the manuscript.

### Conflict of interest statement

The authors declare that the research was conducted in the absence of any commercial or financial relationships that could be construed as a potential conflict of interest.

## Appendix

The individual-based modeling equations assuming 2 planes of division lead to:

(A1a) $$\delta X^{i}=\mu \delta t+\xi \delta W\text{ with }X^{i}=\ln(V^{i})$$

(A1b) $$\begin{array}{c} \text{If }X^{i}\geq X_{m}\sim f_{X_{m}}(x),X^{n+1}\to X^{i}-\text{ln}(4) \end{array}$$

(A1c) $$\begin{array}{c} X^{n+2}\to X^{i}-\text{ln}(4) \end{array}$$

(A1d) $$\begin{array}{c} X^{n+3}\to X^{i}-\text{ln}(4) \end{array}$$

(A1e) $$\begin{array}{c} X^{i}\to X^{i}-\text{ln}(4) \end{array}$$

and the equivalent modified Fokker-Planck equation now reads:

(A2a) $$\begin{array}{l} \frac{\partial p(t,x)}{\partial t}=\underset{\text{cell}\;\text{growth}=\frac{\partial J(t,x)}{\partial x}}{\underset{︸}{\frac{\xi^{2}}{2}\frac{\partial^{2}p(t,x)}{\partial x^{2}}-\mu \frac{\partial p(t,x)}{\partial x}}}+\underset{\text{division}}{\underset{︸}{4f_{X_{d}}(x)Z-f_{X_{m}}(x)Z}}\\ \;-\underset{\text{normalization}}{\underset{︸}{3p(t,x)Z}}\text{ being} \end{array}$$

(A2b) $$\begin{array}{c} Z=\int_{\underline{x}}^{\bar{x}}F_{m}(x)\frac{\partial J(t,x)}{\partial x}dx \end{array}$$

(A2c) $$\begin{array}{c} p(t,\underline{x})=p(t,\bar{x})=0\;\forall t\text{ boundary conditions} \end{array}$$

(A2d) $$\begin{array}{c} p(0,x)=\delta (x-x_{d})\;\forall x\text{ initial conditions} \end{array}$$

## References

[B1] Alonso A. A. Arias-Méndez A. Balsa-Canto E. García M. R. Molina J. I. Vilas C. (2013). Real time optimization for quality control of batch thermal sterilization of prepackaged foods. Food Control 32, 392–403. 10.1016/j.foodcont.2013.01.002

[B2] Alonso A. A. Molina I. Theodoropoulos C. (2014). Modeling bacterial population growth from stochastic single cell dynamics. Appl. Environ. Microbiol. 80, 5241–5253. 10.1128/AEM.01423-14 24928885 PMC4136116

[B3] Amir A. (2014). Cell size regulation in bacteria. Phys. Rev. Lett. 112:208102 10.1103/PhysRevLett.112.208102

[B4] An G. Fitzpatrick B. G. Christley S. Federico P. Kanarek A. Neilan R. M.. (2017). Optimization and control of agent-based models in biology: a perspective. Bull. Math. Biol. 79, 63–87. 10.1007/s11538-016-0225-6 27826879 PMC5209420

[B5] Anastasiadou S. Papagianni M. Filiousis G. Ambrosiadis I. Koidis P. (2008). Pediocin SA-1, an antimicrobial peptide from *Pediococcus acidilactici* NRRL B5627: production conditions, purification and characterization. Bioresour. Technol. 99, 5384–5390. 10.1016/j.biortech.2007.11.015 18093831

[B6] Athale C. A. Chaudhari H. (2011). Population length variability and nucleoid numbers in *Escherichia coli*. Bioinformatics 27, 2944–2948. 10.1093/bioinformatics/btr501 21930671

[B7] Augustin J. C. Ferrier R. Hezard B. Lintz A. Stahl V. (2015). Comparison of individual-based modeling and population approaches for prediction of foodborne pathogens growth. Food Microbiol. 45, 205–215. 10.1016/j.fm.2014.04.006 25500386

[B8] Banwo K. Sanni A. Tan H. (2013). Technological properties and probiotic potential of *Enterococcus faecium* strains isolated from cow milk. J. Appl. Microbiol. 114, 229–241. 10.1111/jam.12031 23035976

[B9] Baranyi J. Roberts T. A. (1995). Mathematics of predictive food microbiology. Int. J. Food Microbiol. 26, 199–218. 10.1016/0168-1605(94)00121-L 7577358

[B10] Cassin M. H. Lammerding A. M. Todd E. C. D. Ross W. McColl R. S. (1998). Quantitative risk assessment for *Escherichia coli* O157:H7 in ground beef hamburgers. Int. J. Food Microbiol. 41, 21–44. 10.1016/S0168-1605(98)00028-2 9631335

[B11] Chandler W. L. Yeung W. Tait J. F. (2011). A new microparticle size calibration standard for use in measuring smaller microparticles using a new flow cytometer. J. Thromb. Haemost. 9, 1216–1224. 10.1111/j.1538-7836.2011.04283.x 21481178

[B12] Deforet M. van Ditmarsch D. Xavier J. B. (2015). Cell-size homeostasis and the incremental rule in a bacterial pathogen. Biophys. J. 109, 521–528. 10.1016/j.bpj.2015.07.002 26244734 PMC4572571

[B13] Egea J. A. Vazquez E. Banga J. R. Martín R. (2009). Improved scatter search for the global optimization of computationally expensive dynamic models. J. Glob. Optim. 43, 175–190. 10.1007/s10898-007-9172-y

[B14] Fahse L. Wissel C. Grimm V. (1998). Reconciling classical and individual-based approaches in theoretical population ecology: a protocol for extracting population parameters from individual-based models. Am. Nat. 152, 838–52. 10.1086/286212 18811431

[B15] Ferrer J. Prats C. López D. Vives-Rego J. (2009). Mathematical modelling methodologies in predictive food microbiology: a SWOT analysis. Int. J. Food Microbiol. 134, 2–8. 10.1016/j.ijfoodmicro.2009.01.016 19217180

[B16] Fishov I. Zaritsky A. Grover N. B. (1995). On microbial states of growth. Mol. Microbiol. 15, 789–794. 7596281 10.1111/j.1365-2958.1995.tb02349.x

[B17] Freedman D. Diaconis P. (1981). On the histogram as a density estimator:L 2 theory. Z. Wahrscheinlichkeitstheor. Verw. Geb. 57, 453–476. 10.1007/BF01025868

[B18] Gangan M. S. Athale C. A. (2017). Threshold effect of growth rate on population variability of *Escherichia coli* cell lengths. R. Soc. Open Sci. 4:160417. 10.1098/rsos.160417 28386413 PMC5367290

[B19] García M. R. Cabo M. L. Herrera J. R. Ramilo-Fernández G. Alonso A. A. Balsa-Canto E. (2017). Smart sensor to predict retail fresh fish quality under ice storage. J. Food Eng. 197, 87–97. 10.1016/j.jfoodeng.2016.11.006

[B20] García M. R. Vilas C. Banga J. R. Alonso A. A. (2008). Exponential observers for distributed tubular (bio)reactors. AIChE J. 54, 2943–2956. 10.1002/aic.11571

[B21] García M. R. Vilas C. Herrera J. R. Bernárdez M. Balsa-Canto E. Alonso A. A. (2015). Quality and shelf-life prediction for retail fresh hake (*Merluccius merluccius*). Int. J. Food Microbiol. 208, 65–74. 10.1016/j.ijfoodmicro.2015.05.012 26058006

[B22] Gardiner C. (2004). Handbook of Stochastic Methods: For Physics, Chemistry & the Natural Sciences. Berlin: Springer.

[B23] Harris L. K. Theriot J. A. (2016). Relative rates of surface and volume synthesis set bacterial cell size. Cell 165, 1479–1492. 10.1016/j.cell.2016.05.045 27259152 PMC4933963

[B24] Higham D. J. (2001). An algorithmic introduction to numerical simulation of stochastic differential equations. SIAM Rev. 43, 525–546. 10.1137/S0036144500378302

[B25] Holt J. G. (1994). Bergey's Manual of Determinative Bacteriology, 9th Edn. Baltimore, MD: Williams & Wilkins.

[B26] Julià O. Comas J. Vives-Rego J. (2000). Second-order functions are the simplest correlations between flow cytometric light scatter and bacterial diameter. J. Microbiol. Methods 40, 57–61. 10.1016/S0167-7012(99)00132-3 10739343

[B27] Kaya T. Koser H. (2009). Characterization of hydrodynamic surface interactions of *Escherichia coli* cell bodies in shear flow. Phys. Rev. Lett. 103:138103. 10.1103/PhysRevLett.103.138103 19905544

[B28] Koch A. L. (1966). Distribution of cell size in growing cultures of bacteria and the applicability of the collins-richmond principle. J. Gen. Microbiol. 45, 409–417. 10.1099/00221287-45-3-409

[B29] Koutsoumanis K. Nychas G.-J. E. (2000). Application of a systematic experimental procedure to develop a microbial model for rapid fish shelf life predictions. Int. J. Food Microbiol. 60, 171–184. 10.1016/S0168-1605(00)00309-3 11016607

[B30] Koutsoumanis K. P. Aspridou Z. (2017). Individual cell heterogeneity in predictive food microbiology: challenges in predicting a “noisy” world. Int. J. Food Microbiol. 240, 3–10. 10.1016/j.ijfoodmicro.2016.06.021 27412586

[B31] Membré J. M. Lambert R. J. W. (2008). Application of predictive modelling techniques in industry: from food design up to risk assessment. Int. J. Food Microbiol. 128, 10–15. 10.1016/j.ijfoodmicro.2008.07.006 18701182

[B32] Métris A. Le Marc Y. Elfwing A. Ballagi A. Baranyi J. (2005). Modelling the variability of lag times and the first generation times of single cells of. I. J. Food Microbiol. 100, 13–19. 10.1016/j.ijfoodmicro.2004.10.004 15854688

[B33] Monteiro J. M. Fernandes P. B. Vaz F. Pereira A. R. Tavares A. C. Ferreira M. T.. (2015). Cell shape dynamics during the staphylococcal cell cycle. Nat. Commun. 6:8055. 10.1038/ncomms9055 26278781 PMC4557339

[B34] Painter P. R. Marr A. G. (1968). Mathematics of microbial populations. Annu. Rev. Microbiol. 22, 519–548. 10.1146/annurev.mi.22.100168.002511 4879521

[B35] Peleg M. Shetty K. (1997). Modeling microbial populations with the original and modified versions of the continuous and discrete logistic equations. Crit. Rev. Food Sci. Nutr. 37, 471–490. 10.1080/10408399709527785 9315435

[B36] Pinho M. G. Kjos M. Veening J. (2013). How to get (a)round: mechanisms controlling growth and division of coccoid bacteria. Nat. Rev. Microbiol. 11, 601–614. 10.1038/nrmicro3088 23949602

[B37] Planas M. Vázquez J. Marqués J. Pérez-Lomba R. González M. Murado M. (2004). Enhancement of rotifer (*Brachionus plicatilis*) growth by using terrestrial lactic acid bacteria. Aquaculture 240, 313–329. 10.1016/j.aquaculture.2004.07.016

[B38] Prats C. Ferrer J. López D. Giró A. Vives-Rego J. (2010). On the evolution of cell size distribution during bacterial growth cycle: experimental observations and individual-based model simulations. Afr. J. Microbiol. Res. 4, 400–407.

[B39] Ray B. (1992). Bacteriocins of starter culture bacteria as food biopreservatives: an overview, in Food Biopreservatives of Microbial Origin, eds Ray B. Daeschael M. A. (Boca Raton, FL: CRC Press), 177–205.

[B40] Robert L. Hoffmann M. Krell N. Aymerich S. Robert J. Doumic M. (2014). Division in *Escherichia coli* is triggered by a size-sensing rather than a timing mechanism. BMC Biol. 12:17. 10.1186/1741-7007-12-17 24580833 PMC4016582

[B41] Ross T. Dalgaard P. Tienungoon S. (2000). Predictive modelling of the growth and survival of Listeria in fishery products. Int. J. Food Microbiol. 62, 231–245. 10.1016/S0168-1605(00)00340-8 11156267

[B42] Sauls J. T. Li D. Jun S. (2016). Adder and a coarse-grained approach to cell size homeostasis in bacteria. Curr. Opin. Cell Biol. 38, 38–44. 10.1016/j.ceb.2016.02.004 26901290 PMC5660915

[B43] Scatassa M. L. Gaglio R. Macaluso G. Francesca N. Randazzo W. Cardamone C.. (2015). Transfer, composition and technological characterization of the lactic acid bacterial populations of the wooden vats used to produce traditional stretched cheeses. Food Microbiol. 52, 31–41. 10.1016/j.fm.2015.06.008 26338114

[B44] Shampine L. F. Reichelt M. W. (1997). The MATLAB ODE suite. SIAM J. Sci. Comput. 18, 1–22. 10.1137/S1064827594276424

[B45] Simpson R. Almonacid-Merino S. F. Torres J. A. (1993). Mathematical models and logic for the computer control of batch retorts: Conduction-heated foods. J. Food Eng. 20, 283–295. 10.1016/0260-8774(93)90069-V

[B46] Standen B. T. Rodiles A. Peggs D. L. Davies S. J. Santos G. A. Merrifield D. L. (2015). Modulation of the intestinal microbiota and morphology of tilapia, *Oreochromis niloticus*, following the application of a multi-species probiotic. Appl. Microbiol. Biotechnol. 99, 8403–8417. 10.1007/s00253-015-6702-2 26115752

[B47] Taheri-Araghi S. (2015). Self-consistent examination of Donachie's constant initiation size at the single-cell level. Front. Microbiol. 6:1349. 10.3389/fmicb.2015.01349 26696971 PMC4672070

[B48] Taheri-Araghi S. Bradde S. Sauls J. T. Hill N. S. Levin P. A. Paulsson J.. (2015). Cell-size control and homeostasis in bacteria. Curr. Biol. 25, 385–391. 10.1016/j.cub.2014.12.009 25544609 PMC4323405

[B49] Trefethen L. N. (2000). Spectral Methods in MATLAB. Philadelphia, PA: Society for Industrial and Applied Mathematics.

[B50] Turner R. D. Ratcliffe E. C. Wheeler R. Golestanian R. Hobbs J. K. Foster S. J. (2010). Peptidoglycan architecture can specify division planes in *Staphylococcus aureus*. Nat. Commun. 1, 1–9. 10.1038/ncomms1025 20975691

[B51] Vande Wouwer A. Saucez P. Vilas C. (2014). Simulation of ODE/PDE Models with MATLAB®, OCTAVE and SCILAB. Scientific and Engineering Applications. Cham: Springer International Publishing.

[B52] Vázquez J. Docasal S. Prieto M. González M. Murado M. (2008). Growth and metabolic features of lactic acid bacteria in media with hydrolysed fish viscera. An approach to bio-silage of fishing by-products. Bioresour. Technol. 99, 6246–6257. 10.1016/j.biortech.2007.12.006 18226525

[B53] Vázquez J. A. Murado M. A. (2008). Mathematical tools for objective comparison of microbial cultures. Application to evaluation of 15 peptones for lactic acid bacteria productions. Biochem. Eng. J. 39, 276–287. 10.1016/j.bej.2007.09.012

[B54] Vázquez J. A. Nogueira M. Durán A. Prieto M. A. Rodríguez-Amado I. Rial D. (2011). Preparation of marine silage of swordfish, ray and shark visceral waste by lactic acid bacteria. J. Food Eng. 103, 442–448. 10.1016/j.jfoodeng.2010.11.014

[B55] Vilas C. Arias-Méndez A. García M. R. Alonso A. A. Balsa-Canto E. (2017). Towards predictive food process models: a protocol for parameter estimation. Crit. Rev. Food Sci. Nutr. [Epub ahead of print]. 10.1080/10408398.2016.1186591 27246577

[B56] Wang P. Robert L. Pelletier J. Dang W. L. Taddei F. Wright A.. (2010). Robust growth of *Escherichia coli*. Curr. Biol. 20, 1099–1103. 10.1016/j.cub.2010.04.045 20537537 PMC2902570

[B57] Wilson W. G. (1998). Resolving discrepancies between deterministic population models and individual-based simulations. Am. Nat. 151, 116–134. 18811412 10.1086/286106

[B58] Zhou J. Ellis A. V. Voelcker N. H. Zambon A. Horowitz M. Bhaya D.. (2010). Recent developments in PDMS surface modification for microfluidic devices. Electrophoresis 31, 2–16. 10.1002/elps.200900475 20039289

[B59] Zwietering M. H. Jongenburger I. Rombouts F. M. van 't Riet K. (1990). Modeling of the bacterial growth curve. Appl. Environ. Microbiol. 56, 1875–1881. 16348228 10.1128/aem.56.6.1875-1881.1990 PMC184525

