# Corrigendum: Stochastic Individual-Based Modeling of Bacterial Growth and Division Using Flow Cytometry

**DOI:** 10.3389/fmicb.2018.00633

**Published:** 2018-03-28

**Authors:** Míriam R. García, José A. Vázquez, Isabel G. Teixeira, Antonio A. Alonso

**Affiliations:** ^1^Bioprocess Engineering Group, Marine Research Institute-Spanish National Research Council (IIM-CSIC), Vigo, Spain; ^2^Group of Recycling and Valorisation of Waste Materials, Marine Research Institute-Spanish National Research Council (IIM-CSIC), Vigo, Spain; ^3^Oceanology, Marine Research Institute-Spanish National Research Council (IIM-CSIC), Vigo, Spain

**Keywords:** individual-based modeling, stochastic modeling, cell cycle, bacterial growth and division, modified Fokker-Planck equation, flow cytometry, coccoid bacteria, predictive microbiology

1. There was a mistake in the writing of the cell growth term in Equation (2a) as published. The correct version appears below.
  (1) $$\frac{\partial p(t,x)}{\partial t}=\underset{\text{cell growth}=\frac{\partial J(t,x)}{\partial x}}{\underset{︸}{\frac{\xi^{2}}{2}\frac{\partial^{2}p(t,x)}{\partial x^{2}}-\mu \frac{\partial p(t,x)}{\partial x}}}+\underset{\text{division}}{\underset{︸}{2f_{X_{d}}(x)Z-f_{X_{m}}(x)Z}}-\underset{\text{normalization}}{\underset{︸}{p(t,x)Z}}$$
2. The same mistake is repeated in equation (A2a). The correct version appears below.
  (2) $$\frac{\partial p(t,x)}{\partial t}=\underset{\text{cell growth}=\frac{\partial J(t,x)}{\partial x}}{\underset{︸}{\frac{\xi^{2}}{2}\frac{\partial^{2}p(t,x)}{\partial x^{2}}-\mu \frac{\partial p(t,x)}{\partial x}}}+\underset{\text{division}}{\underset{︸}{4f_{X_{d}}(x)Z-f_{X_{m}}(x)Z}}-\underset{\text{normalization}}{\underset{︸}{3p(t,x)Z}}\text{ being}$$

The authors apologize for these mistakes. This error does not change the scientific conclusions of the article in any way.

The original article has been updated.

## Conflict of interest statement

The authors declare that the research was conducted in the absence of any commercial or financial relationships that could be construed as a potential conflict of interest.

